# Melatonin Supplementation Lowers Oxidative Stress and Regulates Adipokines in Obese Patients on a Calorie-Restricted Diet

**DOI:** 10.1155/2017/8494107

**Published:** 2017-09-21

**Authors:** Karolina Szewczyk-Golec, Paweł Rajewski, Marcin Gackowski, Celestyna Mila-Kierzenkowska, Roland Wesołowski, Paweł Sutkowy, Marta Pawłowska, Alina Woźniak

**Affiliations:** ^1^Chair of Medical Biology, Ludwik Rydygier Collegium Medicum of Nicolaus Copernicus University, 24 Karłowicza St, 85-092 Bydgoszcz, Poland; ^2^The Tadeusz Browicz Provincial Hospital for Infectious Diseases and Observation, 12 św. Floriana St, 85-001 Bydgoszcz, Poland; ^3^Department of Toxicology, Ludwik Rydygier Collegium Medicum of Nicolaus Copernicus University, 2 dr. A. Jurasza St, 85-089 Bydgoszcz, Poland

## Abstract

Obesity is one of the major global health problems. Melatonin deficiency has been demonstrated to correlate with obesity. The aim of the study was to estimate the effect of melatonin on oxidative stress and adipokine levels in obese patients on a calorie-restricted diet. Thirty obese patients were supplemented with a daily dose of 10 mg of melatonin (*n* = 15) or placebo (*n* = 15) for 30 days with a calorie-restricted diet. Serum levels of melatonin, 4-hydroxynonenal (HNE), adiponectin, omentin-1, leptin, and resistin, as well as erythrocytic malondialdehyde (MDA) concentration and Zn/Cu-superoxide dismutase, catalase, and glutathione peroxidase (GPx) activities, were measured at baseline and after supplementation. Significant body weight reduction was observed only in the melatonin group. After melatonin supplementation, the adiponectin and omentin-1 levels and GPx activities statistically increased, whereas the MDA concentrations were reduced. In the placebo group, a significant rise in the HNE and a drop in the melatonin concentrations were found. The results show evidence of increased oxidative stress accompanying calorie restriction. Melatonin supplementation facilitated body weight reduction, improved the antioxidant defense, and regulated adipokine secretion. The findings strongly suggest that melatonin should be considered in obesity management. This trial is registered with CTRI/2017/07/009093.

## 1. Introduction

Obesity and its medical consequences are major health problems worldwide [1–3]. A body mass index (BMI) over 30 kg/m^2^ is one of the main risk factors for morbidity and mortality from many chronic diseases, including cardiovascular diseases, diabetes mellitus type II, and some types of cancer [3–6]. The problem is growing alarmingly in both genders and all age populations not only in high-income countries but also in the poorer regions of the world [1, 2]. The reduction of obesity prevalence and the development of new strategies for the treatment of obesity and its medical complications are undoubtedly a public health priority. To fight this global pandemic, the mechanisms underlying the pathogenesis of obesity and the related chronic diseases are under intensive investigation.

Excessive adiposity is a multifactorial disorder, generally resulting from the interaction of high dietary energy intake, low physical activity, genetic factors, eating disorders, environmental and socioeconomic factors, and societal influence [7]. Adipose tissue is an organ composed of adipocytes, connective tissue matrix, nerve tissue, stromovascular cells, and macrophages [8]. White adipose tissue (WAT) is a predominant form of fat in adults. In addition to its role as a site of triglyceride storage and fatty acid release, WAT has an important endocrine function and secretes more than six hundred bioactive molecules, the adipokines [9]. Adipokines participate in the regulation of numerous processes, including lipid metabolism, appetite and energy balance, insulin sensitivity, angiogenesis, blood pressure, hemostasis and coagulation, immunity, and inflammation [10]. Hyperplasia and hypertrophy of adipose cells, characteristic of obesity, impair the endocrine action of WAT [11]. This dysfunction manifests as changes in the adipokine profile, including the elevated levels of leptin and resistin and the reduced release of adiponectin and omentin-1 [10, 12]. The disturbed pattern of adipokine secretion is involved in the chronic inflammatory state and in the augmented oxidative stress observed in obesity [12]. Obesity induces numerous proinflammatory changes in immune system, including activation of B and T cells and decline in the population of anti-inflammatory eosinophils [13]. As a result of obesity, blood monocytes are recruited in WAT, where they become polarized to the highly proinflammatory state [13]. Furthermore, the balance between pro- and anti-inflammatory adipokines is then strongly shifted to a pro-inflammatory status [10].

Chronic inflammation, tissue dysfunction, increased plasma levels of glucose and lipids, hyperleptinemia, and decreased antioxidant defense belong to the factors disturbing the oxidant-antioxidant balance in the obese state. During obesity, the generation of reactive oxygen species (ROS) is induced via various biochemical mechanisms, including activation of NADPH oxidases and protein kinase C, oxidative phosphorylation, glyceraldehyde auto-oxidation, and polyol and hexosamine pathways [7, 14]. Moreover, ROS have been found to participate in the body weight control by exerting an effect on hypothalamic neurons, which regulate energy homeostasis [15]. The chronic low-grade inflammation and the excessive amounts of ROS are undeniably involved in the pathogenesis of medical problems related to obesity.

An optimal candidate to diminish the deleterious consequences of excessive adiposity would be an agent that simultaneously exhibits anti-inflammatory and antioxidant properties, as well as having an impact on the adipokine secretion. A molecule which meets these conditions is melatonin (N-acetyl-5-methoxytryptamine). This indoleamine, secreted mainly by pinealocytes, is a derivative of tryptophan. Its synthesis/secretion is highest during the dark phase of the light/dark cycle [16]. Melatonin is an important chronobiotic involved in the regulation of internal biological clock, which organizes seasonal and circadian rhythms [17]. As a chronobiological agent, melatonin also participates in the regulation of metabolism and the energy balance in the organism [18, 19]. Moreover, melatonin may increase the energy expenditure by activating brown adipose tissue [20]. It also preserves mitochondrial functions [16, 21, 22]. A wide range of melatonin physiological functions includes its anti-inflammatory and immunomodulatory actions, as well as its potent direct and indirect antioxidant properties [16, 21, 23]. Many metabolic processes in adipose tissue, including the expression and secretion of some adipokines, are under circadian control [24, 25]. This regulation may be at least partly mediated by melatonin, which could have actions on WAT by means of its membrane receptors or via an action on the sympathetic nervous system [26, 27].

Obesity has been related to the chronic sleep disruption, another epidemic of the industrialized world [28–30]. The modern lifestyle is associated with reduced time of sleep and the excessive use of artificial light; these factors disturb circadian rhythms. The deficiency of melatonin may well contribute to obesity and its complications [19, 30]. Numerous studies in animal models point to the beneficial action of melatonin in the prevention of adiposity [31–36]. There are only limited data concerning the melatonin's effect on obesity in humans [37–39]. The aim of the present study was to determine the effect of melatonin supplementation on the antioxidant status and the levels of circulating adipokines involved in the energy homeostasis in obese human subjects on a calorie-restricted diet.

## 2. Material and Methods

### 2.1. Participants

Thirty volunteers with BMI ≥ 30 kg/m^2^ were included in the study. The participants were evaluated by a standard physical examination and routine clinical laboratory tests. According to the International Classification of Diseases, 10th revision [40], all subjects were diagnosed with obesity due to excess calories (E66.0). The exclusion criteria were metabolic syndrome (according to the WHO criteria) [41], addiction to alcohol and/or tobacco, diabetes mellitus, primary hypertension, ischemic heart disease, history of stroke, renal failure, or other conditions of known free radical etiology. All subjects were made aware of the content of the study and provided informed consent to participate in the research. The study was approved by the Local Ethics Committee at Collegium Medicum, Nicolaus Copernicus University (Bydgoszcz, Poland).

### 2.2. Study Design

The eligible participants with obesity class I or class II [42] were randomly allocated into the melatonin (MEL) or placebo (PL) groups. Patients and investigators were unaware of treatment allocation at all times. The randomization codes remained sealed until after data collection and processing and completion of a masked analysis. During the experimental period, 10 mg melatonin (LE–KAM, Zakroczym, Poland) or placebo (equivalent amount of lactose) was supplied in identical cachets to be taken orally in a single dose for 30 days, 1 hr before bedtime. All participants used a calorie-restricted diet (1000–1200 kcal/day for women and 1400–1600 kcal/day for men). No unexpected adverse events were reported during the study.

The anthropometric measurements were performed twice, at baseline and after 30 days of melatonin/placebo supplementation. Body weight and height of the subjects were measured using a medical weighing scale with a height rod. The accuracy of measurements was 0.1 kg and 0.5 cm, respectively. The weight in kg was divided by the square of the height in m to obtain the BMI value. Waist and hip circumferences were recorded using an anthropometric tape accurate to 0.5 cm. The waist circumference was measured in the horizontal plane directly above the umbilicus, whereas the hip circumference was measured at the largest part of the hips. The waist-hip ratio was calculated as the waist circumference divided by the hip circumference.

Blood samples were collected in the morning (08 : 00 hr) after overnight fasting from the median cubital vein into polypropylene tubes (6 mL) without anticoagulant to obtain serum or in EDTA-containing tubes (9 mL) to obtain plasma and erythrocytes at baseline and after 30 days of melatonin/placebo treatment. All samples were centrifuged (6,000*g* for 10 min at 4°C). The serum and plasma were separated and stored at −80°C for further analysis. Subsequently, the erythrocytes were washed three times with a phosphate-buffered saline (PBS) solution at a ratio of 1 : 3 with a simultaneous centrifugation of the sample after each wash (6,000*g* for 10 min at 4°C). The washed red blood cells were mixed with a PBS solution to obtain erythrocytic suspension with a 50% hematocrit index. The suspension was used to determine the parameters of oxidative stress.

### 2.3. Biochemical Analysis

The commercially available enzyme immune assay kits were used to estimate the serum concentrations of melatonin (enzyme-linked immunosorbent assay kit for melatonin; Cloud-Clone Corp., Houston, TX, USA), 4-hydroxynonenal (HNE) (Human 4-Hydroxynonenal ELISA Kit; Cusabio, College Park, MD, USA), adiponectin (Human Adiponectin ELISA, High Sensitivity; BioVendor, Brno, Czech Republic), leptin (Human Leptin ELISA, Clinical Range; BioVendor, Brno, Czech Republic), resistin (Human Resistin ELISA; BioVendor, Brno, Czech Republic), and omentin-1 (Human Omentin-1 ELISA; BioVendor, Brno, Czech Republic), according to the manufacturers' instructions.

Erythrocytic malondialdehyde (MDA) concentration and the activities of Zn/Cu-superoxide dismutase (SOD-1; EC 1.15.1.1), catalase (CAT; EC 1.11.1.6), and cytosolic glutathione peroxidase (GPx; EC 1.11.1.9) were assayed according to the methods of Buege and Aust [43] in the modification of Esterbauer and Cheeseman [44], Misra and Fridovich [45], Beers and Sizer [46], and Paglia and Valentine [47], respectively. Briefly, the MDA level was expressed as the concentration of thiobarbituric acid-reactive substances (TBARS), measured at 532 nm. The SOD-1 activity measurement was based on the inhibition of adrenaline oxidation to adrenochrome in alkaline environment, which induced a change in the absorbance at 480 nm. The CAT activity was assessed by recording H_2_O_2_ decomposition at 240 nm. The principle of the method for GPx activity measurement was based on recording the decrease in absorbance of NADPH at 340 nm using H_2_O_2_ and reduced glutathione as substrates, in the presence of yeast glutathione reductase and NaN_3_ to inhibit the pseudoperoxidase activity of Hb. The Hb concentration was estimated at 540 nm according to the method of Drabkin [48].

### 2.4. Statistical Analysis

All results are expressed as the means ± S.E.M. Statistical analysis included Student's *t*-test for the comparison of the MEL and PL groups and a paired *t*-test for the comparison of the same group before and after supplementation. The hypothesis of normal distribution was assessed by the Shapiro-Wilk test. In turn, the homogeneity of variances was verified using the Levene's test. Pearson's correlation coefficient was used to quantify the relationship between the parameters measured. The level of significance was set at *P* < 0.05.

## 3. Results

Demographic, anthropometric, and clinical characteristics of the study participants are presented in Table 1. No significant differences were found at baseline between the examined groups. As a result of a calorie-restricted diet, the subject in the MEL and PL groups lost weight, BMI, and waist circumference, but the changes were statistically significant only in the case of body weight in the MEL group (Table 2).

The results of biochemical measurements at baseline and after 30 days of calorie-restricted diet subjects with the melatonin or placebo supplementation are summarized in Table 3. Thirty days of melatonin supplementation resulted in the significant increases in the serum adiponectin and omentin-1 concentrations, as well as the erythrocytic GPx activities. The levels of erythrocytic MDA significantly decreased after melatonin treatment. In the PL group, the serum HNE levels statistically increased, whereas the serum levels of melatonin were significantly reduced after 30 days of a calorie-restricted diet. The remaining parameters did not change in both examined groups.

When data from all subjects were pooled, the interesting correlations were revealed. Statistically significant negative correlations were noted between body weight and adiponectin (*r* = −0.393; *P* < 0.05), BMI and adiponectin (*r* = −0.385; *P* < 0.05), and leptin and omentin-1 (*r* = −0.355; *P* < 0.05). Significant positive correlation was found between BMI and leptin (*r* = 0.492; *P* < 0.001). MDA concentration was negatively correlated with GPx activity (*r* = −0.337; *P* < 0.05), but surprisingly, it was positively correlated with SOD-1 (*r* = 0.358; *P* < 0.05) and CAT (*r* = 0.380; *P* < 0.05) activities.

## 4. Discussion

Taking into account the promising results of animal model studies, the present study was conducted to estimate the effect of a 30-day supplementation of melatonin (10 mg daily) on redox status and circulating adipokines in obese human subjects on a calorie-restricted diet. As mentioned, there exist only a few reports related to the impact of exogenous melatonin on obesity in humans. Koziróg et al. [38] investigated the effect of 5 mg melatonin given daily for 2 months on blood pressure, lipid profile, and oxidative stress in patients with metabolic syndrome. In the study of Chojnacki et al. [37], designed to assess the drug influence on mood, sleep quality, and BMI in overweight/obese postmenopausal women, melatonin (5 mg daily) was administrated in addition to an antidepressant fluoxetine for 24 weeks. By comparison, Mesri Alamdari et al. [39] supplemented 6 mg melatonin daily with a low-calorie diet for 40 days in healthy obese women to evaluate the changes of oxidative stress and the inflammatory state. The present study is the first trial utilizing healthy obese participants of both genders on calorie restriction. It is also worth noting that the dose of melatonin administered in the trial is larger than those in the earlier reports. In animal model studies, melatonin was supplemented to rats at an average dose 0.5–30 mg/kg/day [31–36]. However, in the case of human studies, the safety and possible side effects should be taken under consideration. The dose of 10 mg of the indoleamine per day is twice as large as recommended by the Office for Registration of Medical Products, Medical Devices and Biocidal Products in Poland to treat sleeping problems. Ethical approval of such a dose of melatonin was obtained from the local Ethics Committee. As no adverse effects were reported during the present study, higher doses of hormone may be considered in the subsequent experiments.

A 30-day calorie-restricted diet combined with melatonin supplementation resulted in a statistically significant weight loss, whereas a low-calorie diet per se yielded only a slight reduction in body weight. This is in accordance with the results of many animal studies [32–34, 36]. In these studies, melatonin supplementation reduced body weight or attenuated weight gain in diet-induced obese rats, especially on a high-fat diet. The results are also consistent with the human studies of Chojnacki et al. [37] and Koziróg et al. [38]. However, Mesri Alamdari et al. [39] observed no difference of weight loss degree between women taking melatonin in addition to a low-calorie diet and women on the diet receiving placebo. These apparent differences may depend on a dose of melatonin given, the duration of treatment, and the differences between the groups examined. The effect of exogenous melatonin on weight reduction may be explained by numerous physiologic actions of the indoleamine, including the browning of WAT, the regulation of energy balance and metabolism rate, or chronoregulatory impact on adipocyte functions [19, 20, 22, 30]. In addition, the regulation of appetite and the improvement of sleep quality should be also taken into consideration [30, 37].

Obesity is associated with an altered redox state, including both enhanced reactive oxygen species (ROS) generation and inadequate antioxidant defense [49]. These conditions favor the deleterious processes that lead to obesity-related complications. Weight reduction seems to be a profitable strategy to improve metabolic and cardiovascular risk factors, among others, the proantioxidant status of the organism [50]. Weight loss due to a low-calorie diet and exercise has a positive influence on pro- and antioxidant balance [51–55]. On the contrary, the elevated HNE and the lowered melatonin concentrations, documented in the present study, indicate that a 30-day calorie-restricted diet per se caused an increase of oxidative stress. This may be at least partly a result of inadequate dietary intake of antioxidants [49]. An additional explanation may be the increased blood levels of potentially toxic pollutants released from adipose tissue along with mobilized lipids as subjects lost weight [56, 57]. These toxins, including organochlorine insecticides, induce ROS production [58]. The observed decreased morning melatonin levels support this hypothesis. Undoubtedly, melatonin could be used to neutralize the elevated amounts of ROS [21, 23]. However, it should be noted that a calorie-restricted diet may contain limited amounts of melatonin or its precursor tryptophan [59, 60]. Accordingly, Duggan et al. [61] observed the elevated amounts of fluorescent products of DNA, protein, and lipid oxidation (FOPs) after a 12-month treatment of both reduced calorie diet and reduced calorie diet combined with exercise.

The results of the present study indicate that exogenous melatonin may prevent the rise in oxidative stress in the initial stage of weight loss. When melatonin was supplemented to the subjects, there were no changes in the HNE and melatonin concentrations after 30 days of a low-calorie diet. Exogenous melatonin could be used to counteract the increased oxidative stress accompanying the weight loss. HNE is a major product of nonenzymatic peroxidation of polyunsaturated fatty acids [62]. HNE is known to have mutagenic, cytotoxic, and carcinogenic effects, but in the physiologic levels, it may exert a lipohormetic action, enabling cells to adapt to stress-induced mediators [63]. High levels of HNE are strongly associated with the development of obesity and its complications, leading to pathogenic cellular changes and promoting numerous disease processes [64]. Herein, exogenous melatonin stabilized the HNE level in the subjects on a low-calorie diet and prevented a decrease in morning endogenous melatonin concentrations. Moreover, the erythrocytic MDA level significantly decreased and GPx activity increased as a result of melatonin administration in addition to a low-calorie diet; thus, the redox state of these subjects was improved. Mesri Alamdari et al. [39] observed a significant drop in MDA levels in obese women on a low-calorie diet with melatonin supplementation, but the total antioxidant capacity remained unchanged. Similarly, a 2-month melatonin supplementation caused a reduction in TBARS concentration and a rise in SOD activity in the metabolic syndrome patients [38]. In other studies, melatonin supplementation improved the proantioxidant state in patients suffering from diseases linked to obesity, including diabetes mellitus [65], hypertension [66], and chronic obstructive pulmonary disease [67]. The direct and indirect antioxidant properties of melatonin and its metabolites are well documented [18, 21, 23]. It was shown in numerous in vivo and in vitro studies that melatonin scavenges oxygen- and nitrogen-reactive species [21, 23, 68, 69], protects against lipid peroxidation [70, 71], and stimulates mRNAs and activities of antioxidant enzymes [72, 73].

Positive correlations between erythrocytic MDA concentration and the activities of SOD-1 and CAT are important observations in the present study. In the early stages of obesity, compensatory mechanisms stimulate the activities of antioxidant enzymes to counteract the augmented synthesis of ROS, but they are insufficient to prevent oxidative damage [14, 49]. The present study included obese people with no known health complications; thus, we presume that their antioxidant defense was activated to limit oxidative stress, but it was insufficient to totally prevent oxidative damage.

Excessive adiposity is a condition characterized by a severe dysfunction of WAT, including the alterations of tissue composition and the modifications of its endocrine function [74]. The disruption of adipokine secretion pattern seems to be pivotal in the pathophysiology of obesity-induced metabolic dysfunctions, including metabolic syndrome, diabetes mellitus type II, and atherosclerosis [10, 12]. This disruption may be associated with the differences in adipokine secretion found between the subcutaneous and visceral WAT depots [5, 75]. In obesity, the characteristic predominance of visceral WAT is a factor that increases mortality and risk for obesity-related disorders [75]. Adipokines involved in the regulation of energy production and expenditure seem to be of special importance. Adiponectin, omentin-1, leptin, and resistin, measured in the present study, participate in these processes.

Adiponectin is a hormone strongly involved in the regulation of lipid and glucose metabolism. It improves insulin sensitivity, regulates appetite and energy expenditure, and exerts anti-inflammatory, antiproliferative, and antiapoptotic actions [76]. Adiponectin is produced in higher amounts in subcutaneous than in visceral WAT [77]. In the present study, a negative correlation was found either between body weight and adiponectin and between BMI and adiponectin. This is in agreement with other studies, which reported a negative correlation of circulating adiponectin and BMI, metabolic syndrome, insulin resistance, and diabetes mellitus type 2 [78–80]. Interestingly, the circadian rhythm of adiponectin expression was observed to be attenuated in obesity [81], whereas weight loss was found to be associated with a rise in plasma adiponectin and the recovery of its secretion pattern [78, 82].

In the present study, a 30-day low-calorie diet resulted in no change of the circulating morning levels of adiponectin, whereas the supplementation of melatonin significantly increased the concentration of this adipokine. Gonciarz et al. [83] also found a positive effect of exogenous melatonin (5 mg twice a day) on the levels of circulating adiponectin in overweight patients with nonalcoholic steatohepatitis. Moreover, it was demonstrated that adiponectin positively correlated with circadian amplitude of melatonin secretion in healthy women, but not in the metabolic syndrome women [84]. Many animal model studies confirmed the stimulatory effect of melatonin treatment on circulating adiponectin [31, 32, 85], but there exist some inconsistencies in other experiments [76]. Several mechanisms may participate in melatonin's influence on adiponectin secretion, including the impact of indoleamine on adiponectin signaling pathways, its antioxidant and anti-inflammatory properties, mitochondrial function improvement, and changes in other adipokine levels [23, 76, 86, 87].

Omentin-1 is another insulin-sensitizing and anti-inflammatory adipokine [10, 74]. It is synthesized mainly in visceral WAT and was found to enhance insulin-stimulated glucose uptake in adipocytes [88, 89]. No circadian rhythm of omentin-1 secretion has been observed in humans [90]. Omentin-1 was demonstrated to be decreased in obesity and inversely correlated with BMI, insulin resistance, and metabolic syndrome [91–93]. Moreno-Navarrete et al. [94] observed elevated omentin-1 levels after hypocaloric weight loss. However, in the present study, the elevated serum omentin-1 concentrations were found only in the low-calorie diet group supplemented with melatonin. As known so far, this is the first report concerning the influence of melatonin on circulating omentin-1. The possible mechanisms of melatonin action on omentin-1 may be corresponding to its effect on the levels of adiponectin.

Both adiponectin and omentin-1 were found to improve insulin sensitivity [74, 76]. Increased levels of these adipokines after melatonin supplementation, observed in the present study, are consistent with the research of McMullan et al. [95]. They found a positive association of high-nocturnal-melatonin secretion with greater insulin sensitivity and a lower prevalence of insulin resistance in healthy young women. This relationship may be at least partly explained by the influence of melatonin on the adiponectin and omentin-1 secretion. These results strongly support the use of melatonin in the treatment of obesity and prevention of its complications, especially diabetes mellitus. It should be mentioned that Rubio-Sastre et al. [96] observed impaired glucose tolerance in healthy young women after morning and evening acute melatonin administration. Considering numerous animal model studies [76], it should be emphasized that prolonged supplementation of melatonin, mimicking the conditions of normal sleep, is necessary to obtain the beneficial effects of the hormone in the treatment and prevention of obesity. Thus, a physiological pattern of melatonin supplementation should be absolutely considered, avoiding acute administration.

Leptin- and resistin-exhibited elevated levels are positively correlated with obesity and its complications [7, 12, 88]. Leptin is a hormone produced mainly in adipocytes in a circadian manner [80]. Subcutaneous WAT depots secrete larger amounts of leptin than visceral WAT [97]. Leptin is strongly involved in the regulation of food intake and energy balance [76]. However, in obesity, elevated levels of the hormone fail to regulate the body weight due to leptin resistance [98]. According to other reports, a significant positive correlation was found between BMI and leptin in the present study. Moreover, similar to the de Souza Batista et al. study [91], leptin and omentin-1 were negatively correlated, indicating the regulatory relationship between synthesis and secretion of these two adipokines. In most animal model studies, exogenous melatonin was found to decrease the circulating leptin levels [31, 33, 36]. In human studies, the results are inconsistent [83, 99, 100]. Herein, leptin levels remained unchanged after a 30-day low-calorie diet in both melatonin and placebo groups.

Similar results were obtained in the case of resistin. Resistin is a proinflammatory adipokine, which is associated with the development of insulin resistance [12, 101]. The results of studies concerning the possible links between resistin and obesity are rather disputable [88], but some reports demonstrated reduced resistin levels following the loss of body weight in humans [102]. Similar to the results of the present study, Gonciarz et al. [83] found no change of circulating resistin after melatonin supplementation. However, in the Favero et al. study [103], melatonin supplementation reduced resistin levels in obese (*ob/ob*) mice. The links between resistin and obesity need further investigations.

## 5. Conclusions

The positive impact of exogenous melatonin on weight reduction, observed in the present study, strongly supports that administration of this agent may be a useful adjunct in obesity treatment. In addition, melatonin significantly reduced oxidative stress and regulated the circulating adipokines beneficial for energy homeostasis in obese subjects on a low-calorie diet. Thus, exogenous melatonin may facilitate the health improvement during obesity management. These results confirm that melatonin supplementation, mimicking the conditions of normal sleep, could have a desirable effect in humans during dietary weight loss.

The present study has some limitations. The small number of participants is a limiting factor; thus, it may be treated as a pilot study. However, to the best of the authors' knowledge, it is the first report related to the effects of melatonin supplementation on human obesity with such large doses of the hormone. Another limitation is the study design—the blood samples were taken only once a day, in the morning. It was sufficient to observe the changes in the circulating levels of the selected adipokines. Nevertheless, in the case of adipokines with the circadian pattern of secretion, it would be interesting to examine their maximum release after melatonin supplementation. Undoubtedly, the results of the present study are very promising but raised new questions requiring further research.

## Figures and Tables

**Table 1:** Demographic, anthropometric, and clinical characteristics at baseline of the study participants, according to treatment with melatonin (MEL group) or placebo (PL group).

Parameter	MEL group	PL group	*P* value
*n* (male/female)	15 (5/10)	15 (5/10)	
Age (yrs)	37.7 ± 3.40	36.3 ± 4.18	0.79
Body weight (kg)	113.6 ± 4.74	114.4 ± 7.31	0.93
Height (m)	1.74 ± 0.024	1.72 ± 0.025	0.74
BMI (kg/m^2^)	37.8 ± 1.51	38.2 ± 1.94	0.85
Waist circumference (cm)	112.4 ± 3.50	112.6 ± 4.13	0.97
Hip circumference (cm)	121.5 ± 2.78	123.4 ± 4.42	0.71
Waist-hip ratio	0.93 ± 0.04	0.92 ± 0.04	0.82
SBP	124.2 ± 2.32	125.0 ± 4.36	0.87
DBP	75.8 ± 1.73	76.7 ± 2.35	0.78
Total cholesterol (mg/dL)	196.7 ± 5.99	196.6 ± 7.02	0.99
Glucose (mg/dL)	94.0 ± 1.90	99.9 ± 4.70	0.15

Each value is mean ± S.E.M. BMI: body mass index; SBP: systolic blood pressure; DBP: diastolic blood pressure.

**Table 2:** Anthropometric parameters in the obese subjects on calorie-restricted diet after 30 days of melatonin (MEL; 10 mg/day) or placebo (PL) administration.

Parameter	MEL group (*n* = 15)	The percentage change	*P* value^∗^	PL group (*n* = 15)	The percentage change	*P* value^∗^
Body weight (kg)	105.9 ± 6.43	7%	0.039	109.8 ± 8.57	4%	0.69
BMI (kg/m^2^)	35.5 ± 1.52	6%	0.42	36.3 ± 2.22	5%	0.54
Waist circumference (cm)	107.1 ± 4.60	5%	0.38	107.2 ± 4.74	5%	0.41

Each value is mean ± S.E.M. BMI: body mass index. ∗ within-group comparison: baseline versus day 30.

**Table 3:** Effects of 30 days of melatonin (MEL; 10 mg/day) or placebo (PL) administration on the biochemical parameters in the obese subjects on calorie-restricted diet.

Parameter		MEL group (*n* = 15)			PL group (*n* = 15)		
		Baseline	After treatment	*P* value	Baseline	After treatment	*P* value
Serum	Melatonin (ng/L)	30.7 ± 6.37	33.7 ± 4.96	0.44	34.0 ± 2.55	25.0 ± 1.79	0.014
	HNE (*μ*g/L)	8.19 ± 1.36	8.48 ± 1.20	0.51	7.86 ± 0.68	18.4 ± 6.1	0.044
	Adiponectin (mg/L)	2.82 ± 0.17	3.46 ± 0.18	0.029	3.17 ± 0.36	3.05 ± 0.56	0.70
	Omentin-1 (*μ*g/L)	380.1 ± 16.2	468.9 ± 16.7	0.0044	383.2 ± 20.8	396.4 ± 30.4	0.82
	Leptin (*μ*g/L)	33.1 ± 4.85	36.6 ± 5.76	0.70	36.9 ± 3.36	32.1 ± 6.86	0.58
	Resistin (*μ*g/L)	4.70 ± 0.42	4.45 ± 0.54	0.97	5.36 ± 0.41	5.16 ± 0.60	0.86
Erythrocyte	MDA (nmol/g Hb)	34.3 ± 2.44	24.5 ± 2.16	0.042	30.1 ± 2.89	27.4 ± 2.19	0.65
	SOD-1 (IU/g Hb)	747.7 ± 19.4	762.0 ± 54.4	0.29	697.0 ± 16.7	687.9 ± 32.0	0.94
	CAT (10^4^ × IU/g Hb)	68.5 ± 2.94	64.7 ± 4.23	0.66	63.1 ± 2.89	65.0 ± 4.52	0.61
	GPx (IU/g Hb)	5.49 ± 0.48	7.92 ± 0.56	0.0049	6.19 ± 0.58	7.19 ± 0.34	0.29

Each value is mean ± S.E.M. HNE: 4-hydroxynonenal; MDA: malondialdehyde; SOD-1: Zn/Cu-superoxide dismutase; CAT: catalase; GPx: glutathione peroxidase.
